# Perception of Dutch vowels by Cypriot Greek listeners: To what extent can listeners’ patterns be predicted by acoustic and perceptual similarity?

**DOI:** 10.3758/s13414-023-02781-7

**Published:** 2023-09-22

**Authors:** Georgios P. Georgiou, Dimitra Dimitriou

**Affiliations:** 1 Department of Languages and Literature, University of Nicosia, Nicosia, Cyprus; 2 University of Nicosia Phonetic Lab, Nicosia, Cyprus; 3 University of Central Lancashire Cyprus, Larnaca, Cyprus

**Keywords:** Speech perception, Phonology, Psychoacoustics

## Abstract

There have been numerous studies investigating the perception of non-native sounds by listeners with different first language (L1) backgrounds. However, research needs to expand to under-researched languages and incorporate predictions conducted under the assumptions of new speech models. This study aimed to investigate the perception of Dutch vowels by Cypriot Greek adult listeners and test the predictions of cross-linguistic acoustic and perceptual similarity. The predictions of acoustic similarity were formed using a machine-learning algorithm. Listeners completed a classification test, which served as the baseline for developing the predictions of perceptual similarity by employing the framework of the Universal Perceptual Model (UPM), and an AXB discrimination test; the latter allowed the evaluation of both acoustic and perceptual predictions. The findings indicated that listeners classified each non-native vowel as one or more L1 vowels, while the discrimination accuracy over the non-native contrasts was moderate. In addition, cross-linguistic acoustic similarity predicted to a large extent the classification of non-native sounds in terms of L1 categories and both the acoustic and perceptual similarity predicted the discrimination accuracy of all contrasts. Being in line with prior findings, these findings demonstrate that acoustic and perceptual cues are reliable predictors of non-native contrast discrimination and that the UPM model can make accurate estimations for the discrimination patterns of non-native listeners.

## Introduction

Although newborns are able to distinguish phonetic contrasts in a great number of human languages, even if they had never heard them before, this ability declines after the age of 6–12 months (Cheour et al., 1998; Eimas et al., 1987; Kuhl et al., 1992; Werker & Tees, 1983). It has been proposed that this decline from infancy to adulthood is an outcome of continuous exposure to a particular language, which alters the way non-native speech sounds are perceived (Iverson et al., 2003). As a consequence, listeners of a non-native language struggle to distinguish sounds that are not present in their first language (L1) phonological system (Bohn & Munro, 2007; Strange, 1995); this capacity can be reversed to some extent through phonetic training (e.g., see Georgiou 2021a, 2022a). For example, Spanish listeners usually fail to discriminate English /i – ɪ/ since both vowels are assimilated to Spanish /i/ (Cebrian, 2019; Morrison, 2008). In contrast, German listeners do not have significant difficulties in mastering this English contrast since the German vowel system contains a vowel contrast that is an articulatorily and acoustically close instance of English /i – ɪ/ (Llompart & Reinisch, 2019).

A previous body of work has demonstrated that the size and complexity of the L1 and L2 phonological systems determine listeners’ perceptual abilities in the L2 sounds (e.g., Escudero et al. 2014; Fox et al., 1995; Georgiou et al., 2020; Iverson & Evans, 2007). Iverson and Evans (2007) found that speakers of German and Norwegian, two languages with a larger and more complex vowel system than English, had better perceptual abilities than speakers of Spanish, a language with a smaller and less complex vowel system than English. Nevertheless, evidence from other studies shows that smaller and less complex L1 phonological systems do not always lead to perceptual difficulties (e.g., Alispahic et al., 2014; Alispahic et al., 2017; Elvin et al., 2014). For instance, Elvin et al. (2014) concluded that there is no advantage for Australian English listeners in discriminating the Brazilian Portuguese vowels in comparison to Spanish speakers, although Australian English has a larger vowel inventory than Brazilian Portuguese and Spanish has a smaller vowel inventory. It seems that the consideration of the size and complexity of the L1-L2 phonological inventories alone cannot always predict perceptual abilities in the non-native language.

Several theoretical models assume either explicitly or implicitly that acoustic-phonetic similarity between native and non-native sounds can predict the perceptual patterns of the latter sounds (Escudero 2009; Flege, 1995; Georgiou, 2021b). Escudero and Boersma (2003) argued that when humans perceive speech, they integrate the various auditory dimensions they hear in a manner that mirrors the way those dimensions are combined during speech production. They pointed out that this integration is governed by the “optimal perception hypothesis,” which posits that a listener will prefer auditory dimensions that effectively distinguish sounds in the production of their L1 (Escudero, 2009). Essentially, this means that listeners use their knowledge of L1 speech sounds to guide their perception of speech, relying on auditory dimensions that are most useful in distinguishing between relevant sounds in their L1. Given that the preference for acoustic cues differs across different varieties, the optimal perception of both L1 and the target language can be achieved through a comprehensive acoustic description of both languages’ acoustic features. This description would allow for an explanation of how listeners perceive the target language sounds. In addition, the so-called “full copying hypothesis” suggests that at the initial stage of L2 acquisition, learners establish a copy of their existing L1 perception grammar to perceive the non-native sounds (Elvin & Escudero, 2019); therefore, they initially rely on their L1 to map unfamiliar sounds. So, acoustic similarity between L1 and non-native sounds, which can be roughly defined as the phonetic distance that separates an L1 sound from a target language sound, can be used as a reference for predicting the non-native speech perception patterns.

Different methodologies have been used to calculate cross-linguistic acoustic similarity, with the goal of predicting the mapping of non-native sounds to the speakers’ L1 vowel system and the discrimination of non-native sound contrasts. For example, many studies have employed *Euclidean Distances* (e.g., Elvin et al., 2014; Georgiou et al., 2020), reporting that these values provide accurate perceptual predictions. *Linear Discriminant Analysis* (LDA) (Klecka, 1980) is a machine-learning classifier that aims to identify a linear transformation maximizing the separation between different classes in the reduced-dimensional space, thus improving the accuracy of classification (Park & Park, 2008). The role of LDA in cross-linguistic speech perception is to assess how well target language sounds fit with the center of gravity of the input corpus tokens, providing a predicted estimation of how each sound is mapped to the speakers’ L1 categories (Elvin et al., 2021). Data is split into training and testing subsets, which include acoustic measures from speech samples such as formant frequencies, durations etc. After training the L1 model using the extracted measures, the same measures of non-native sounds from the testing subset are supplied to the model in order to calculate the proportion of their categorization to L1 categories. The confusion matrix can be used to predict the discrimination accuracy of non-native sound contrasts by employing the theoretical framework of a particular speech model. LDA has been used in the past in speech perception studies for the calculation of cross-linguistic acoustic similarity. For example, Gilichinskaya and Strange (2010) investigated the perceptual assimilation of American English vowels to the L1 categories of inexperienced Russian listeners. The results indicated that the algorithm predicted modal assimilation responses for all but one vowel, demonstrating that acoustic similarity is a good predictor of non-native sound categorization. Similarly, in a more recent study, Georgiou (2023a) used LDA to assess whether acoustic similarity could estimate the classification of L2 English vowels as Cypriot Greek L1 categories. The results verified the model’s predictions as the majority of L2 vowels classified with the highest proportion were predicted with success. Other studies provide further support for these findings (e.g., Elvin et al., 2021).

Among the most widely used models in cross-linguistic speech perception is the Perceptual Assimilation Model (PAM) (Best, 1995). PAM aims to predict the discrimination of particular non-native sound contrasts by listeners with little or no experience in the target language. The model suggests that phonological and articulatory-phonetic similarity between two non-native sounds can estimate how these sounds will be discriminated. It proposes six types of assimilations of non-native sounds to L1 phonological categories: Two category (TC) assimilation in which both sounds are assimilated to two different L1 categories (excellent discrimination), Single Category (SC) assimilation in which the two sounds are assimilated to the same L1 category (poor discrimination), Category Goodness (CG) assimilation in which one sound is assimilated as a good phonetic exemplar to an L1 category, while the other as a bad exemplar to the same category (moderate to very good discrimination), Uncategorized – Categorized (UC) assimilation in which one sound is assimilated to an L1 category, while the other does not (very good discrimination), Uncategorized-Uncategorized in which both sounds are not perceived as exemplars of an L1 category (poor to very good discrimination), and Non-assimilable (NA) in which both sounds are perceived as nonspeech sounds (good to very good discrimination). L2LP (Escudero, 2009) is another highly cited speech perception model that provides predictions about the discrimination of non-native contrasts by naïve learners. The predictions rely on cross-linguistic acoustic similarity and are developed using measures such as Euclidean Distances and LDA. Rather than assimilation patterns, it proposes three different learning scenarios, which can estimate the discrimination accuracy of non-native sound contrasts. The Similar Scenario (similar to the TC assimilation of PAM) occurs when two sounds are mapped to two different L1 phonological categories. The New Scenario takes place when two sounds are mapped to a single L1 category (similar to the SC assimilation of PAM). The Subset Scenario occurs when one or both sounds are equated to two or more L1 categories. The Similar Scenario exhibits the most accurate discrimination, followed by the Subset Scenario (only if there is no perceptual overlap), the New Scenario, and finally the Subset Scenario, but only if both non-native sounds are mapped to the same subset of L1 categories.

The *Universal Perceptual Model* (UPM) (Georgiou, 2021b) has been developed to account for the difficulties of listeners/speakers regarding the discrimination of non-native sound contrasts under the assumption that there is a universal capability to perceive speech sounds during the lifespan. The model supports that speech sounds are perceptual in nature, constraining the perception of *phonetic* categories extracted from the speech signal. UPM establishes predictions regarding the classification of non-native sounds in terms of L1 categories and the discrimination of two non-native sounds based on cross-linguistic acoustic similarity using machine-learning algorithms such as LDA. The results of the perceptual classification test, which refers to the actual classification of non-native sounds by listeners/learners of the target language, is used to develop the predictions for the discrimination accuracy of particular non-native sound contrasts according to the framework of UPM. Therefore, the model supports the connection between acoustic similarity and speech perception as emerges from machine-learning algorithms and classification and sound contrast discrimination as emerges from perceptual tasks completed by humans, just like other important models. UPM indicates that sound contrast discrimination is determined by the *overlap degree* between the two contrast members, which depends on how closely the two members are perceptually associated with each other based on their classification in terms of one or more L1 sounds. Specifically, the degree of overlap is determined by observing the classification proportions of each of the two non-native vowels in terms of one or more L1 categories. Crucially for UPM, only above chance classifications matter, that is, non-native sounds classified with a proportion above a chance score, which is determined by dividing the total number of responses by 1.00 (or 100%). UPM proposes three types of overlap between two non-native sounds: complete, partial, and no overlap. Completely overlapping contrasts share the same above chance responses or the same set of above chance responses, partially overlapping contrasts share at least one above chance response, and no overlapping contrasts do not share any above chance responses.

**Fig. 1:**
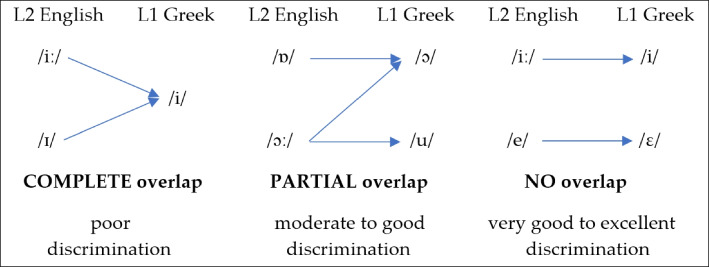
The overlapping degrees of the Universal Perceptual Model (UPM)

For example, if the chance score is 0.20 (or 20%) and if non-native /i/ is classified with a proportion of 0.90 as L1 /i/ and non-native /ɪ/ is classified with a proportion of 0.85 as L1 /i/, then both non-native sounds comprise above chance responses (≥ 0.20) and the contrast is completely overlapping since the two non-native vowels are both classified to a great extent as the same L1 category. However, if non-native /i/ is classified with a proportion of 0.90 as L1 /i/ and non-native /ɪ/ is classified with a proportion of 0.70 as L1 /i/ and with 0.30 as L1 /ε/, then both non-native sounds share only one above chance response (that is, the classification of both non-native vowels as L1 /i/), thereby forming a partially overlapping contrast. Nonoverlapping contrasts are the most discriminable followed by partially and completely overlapping contrasts. However, in completely overlapping contrasts, the discrimination accuracy may be comparable to that of the partially overlapping contrasts if listeners are able to perceive phonetic distance between two non-native sounds, which is usually determined by measuring the difference between the goodness-of-fit ratings of the two responses in the classification test. Figure 1 shows an example of the overlapping degrees of UPM.

This study aims to investigate the perception of Dutch vowels by Cypriot Greek listeners using the theoretical framework of UPM. The vowel systems of Cypriot Greek and Dutch differ to a great extent. Cypriot Greek has the five vowel qualities /i ε ɐ ɔ u/; note that a more generic representation includes the qualities /i e a o u/. There are not any length distinctions, but stressed vowels tend to be longer than unstressed vowels (Georgiou & Themistocleous, 2021). The Dutch vowel system is more complex than the Cypriot Greek system including 12 monophthongs (without schwa). Moulton (1962) distinguishes between the five lax or short /ɪ ɛ ʏ ɑ ɔ/ and the seven tense or long vowels /i y a u e o ø/. Length distinction is considered as part of the syllable rather than a phonological feature (Booij, 1995). There are only very few studies regarding the perception of non-native vowels by Cypriot Greek speakers. For example, Georgiou (2019) examined the perception of English vowels by Cypriot Greek children with low and high proficiency in English. The results showed that English vowels /iː ɪ/, /e ɜː/, /æ ʌ ɑː/, /ɒ ɔː/, /ʊ uː/ were mostly assimilated to Greek phonological categories /i/, /e/, /a/, /o/ and /u/ respectively for children of both proficiency levels. Also, children struggled to discriminate particular English sound contrasts: /iː – ɪ/ and /e – ɜː/ could be discriminated only in a moderate manner, while /æ – ʌ/ and /ɒ – ɔː/ yielded poor discrimination. Georgiou (2021b) assessed the classification of Italian vowels in terms of Cypriot Greek categories and the ability of Cypriot Greek speakers to discriminate pairs of Italian vowel contrasts. The study employed the theoretical framework and predictions of UPM. It was found that Italian vowels /i/, /e/, /ε/, /a/, /o/, /ɔ/, /u/ were classified as above chance responses in terms of Cypriot Greek cardinal vowels /i/, /i e/, /e/, /a/, /u/, /o/, /u/ respectively. The non-overlapping /ɔ – o/ contrast was discriminated well, the partially overlapping /i – e/ and /e – ε/ contrasts were discriminated to a moderate extent and the completely overlapping /o – u/ contrast was discriminated poorly. The results confirmed the predictions of UPM concerning the discriminability of sound contrasts based on their overlapping degree. In another study, Georgiou (2022b) found that both cross-linguistic acoustic similarity and UPM could predict the accuracy of the challenging English /iː – ɪ/ vowel contrast as discriminated by Cypriot Greek speakers.

A second aim of this study is to assess the capacity of the LDA model in predicting the classification/discrimination of non-native sounds based on cross-linguistic acoustic similarity and the ability of the UPM model to make accurate empirical predictions about the discrimination accuracy of non-native sound contrasts based on perceptual similarity. To better understand the acquisition of non-native speech, research needs to include under-researched sets of languages such as Cypriot Greek and Dutch. This is among the first studies that examine the perception of Dutch vowel contrasts by speakers of any Greek variety; for another study examining the perception of other Dutch contrasts by Standard Modern Greek and Cypriot Greek listeners, see Georgiou (2023b). Dutch was chosen since it contains a large and more complex vowel system compared to Cypriot Greek and therefore speakers of the latter variety will experience difficulties in accurately perceiving particular Dutch vowels. It also contains vowel qualities of which the perceptual categorization by Greek speakers has not been investigated in previous studies (e.g., /ʏ/, /ø/, /y/).

The study’s protocol is based on a production and a perception study. In the production study, Cypriot Greek and Dutch speakers produced their L1 vowels and their speech patterns were analyzed using speech processing software. The output of the Cypriot Greek speakers was used to train a machine-learning LDA model, and the output of Dutch speakers was fed into the trained model to generate predictions about the classification of Dutch vowels in terms of listeners’ L1 categories. In the perception study, listeners classified the Dutch vowels in terms of L1 categories and discriminated particular Dutch vowel contrasts using an AXB test. The classification test helped us evaluate the predictions of the LDA model, which provided classification data based on the vowels’ acoustic features (acoustic similarity), and the predictions of UPM, which rely on the overlapping degree of the sound contrast members as reported by the classification test (perceptual similarity).

## Production study

### Methodology

#### Participants

A total number of 32 speakers participated in the study. Twelve participants aged 20–45 years (*M*_age_ = 33, *SD* = 7.9) were Cypriot Greek speakers. They were born and raised in Cyprus and originated from moderate-income families. Their language development was typical and they never experienced any hearing or other cognitive issues. The listeners did not have knowledge of Dutch. Twenty participants were Dutch speakers. The output of these speakers was obtained from the database of Van der Harst (2011), which includes acoustic measurements of Dutch vowels produced by 160 Dutch high school teachers. We selected productions from 20 speakers with an age range of 22–40 years, who belonged to the Netherlandic community. All participants were females.

#### Stimuli

The stimuli of the production test undertaken by the Cypriot Greek listeners consisted of the five Cypriot Greek vowels /i ε ɐ ɔ u/. Vowels were embedded in a /pVs/ context (V = vowel) and were part of the carrier phrase ‘Léne <target word> tóra’ (‘they say <target word> now’). The Dutch stimuli included the Dutch monophthongs /a ɑ i u ɪ ɔ ɛ ʏ o e ø y/. These vowels were embedded in monosyllabic words before coda [s] with the exception of /y/, which was embedded before coda [t] as this vowel does not occur before /s/ besides proper names. The words were part of the carrier phrase “Hoor je <target word>”. While the target words were in a phrase-final position in Dutch and a phrase-medial position in Cypriot Greek, no impact is expected on the classification results. This is because some additional analyses we ran indicated no important differences between the productions in the two conditions. Specifically, five female adult Cypriot Greek speakers produced their native vowels in both a phrase-medial and phrase-final position. The analyses were conducted using linear mixed-effects models in R with *F1, F2, F3*, and DURATION as dependent variables, VOWEL and CONDITION as fixed factors and PARTICIPANTS as a random factor. The findings showed no significant effect of CONDITION on *F1, F2*, and *F3*, while there was a significant effect on DURATION. However, a Tukey posthoc test revealed that these differences concerned only two out of five vowels. So, the differences were minimal. 

#### Procedure

The Cypriot Greek listeners performed the production test individually in quiet rooms. They were instructed to appropriately sit in front of a PC monitor and repeat the carrier phrases presented through Microsoft PowerPoint as if speaking to a friend. They produced a total number of 240 items (5 vowels × 4 repetitions × 12 speakers) and the output was recorded using a professional audio recorder at a 44.1 kHz sampling rate. The stimuli were randomized for each participant. The output of Dutch speakers was retrieved from the database of Van der Harst (2011). Speakers produced a total number of 240 items (12 vowels × 20 speakers) and all values were measured at the midpoint.

**Fig. 2:**
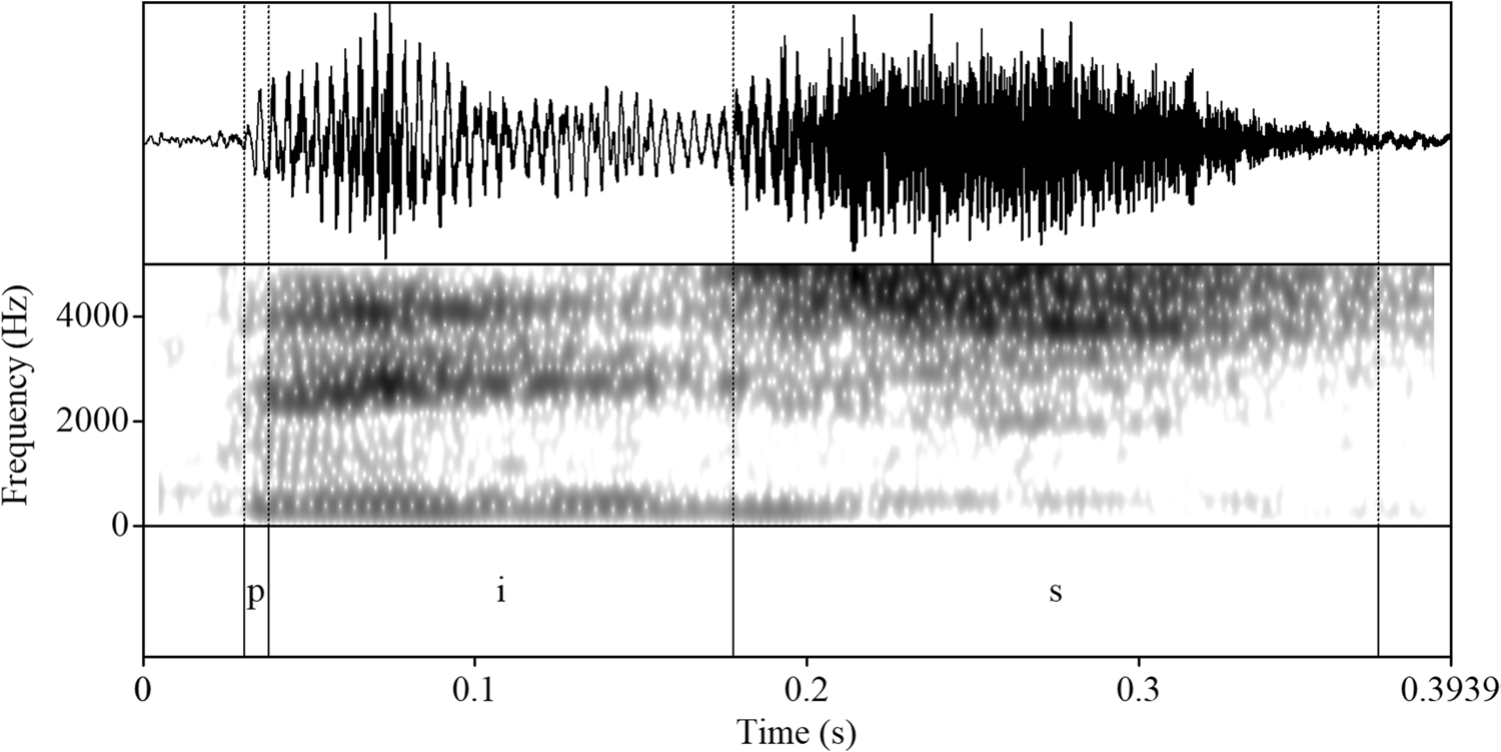
Example of the segmentation process of Cypriot Greek vowel /i/ (/pVs/ context). The upper tier shows the waveform, while the middle tier shows the spectrogram with consonant and vowel boundaries (dashed lines)

The target words from the Cypriot Greek speakers’ output were isolated and sent to Praat (Boersma & Weenink, 2023) for speech analysis. The visual inspection of spectrograms and waveforms based on identifiable acoustic landmarks helped us measure the boundaries of each vowel to extract formant frequencies and vocalic duration. To generate all tracks, the length of windows was set at 0.025 ms, the pre-emphasis at 50 Hz, and the spectrogram view range at 5,500 Hz, with a formant ceiling of 5,500 Hz, suitable for average adult female speakers. For the measurement of formant frequencies, the starting point of vowels’ acoustic analysis was regarded as the end of the burst of the preceding stop consonant /p/ and the onset point of V. The last point of vowels’ acoustic analysis was regarded as the end of periodicity of V as shown in the waveform and the formant structure as shown in the spectrogram (i.e., the acoustic energy concentrated in specific frequency regions) and the onset point of the second consonant /s/. Formants were measured through visual inspection of the spectrogram at their midpoint, where vowels exhibit the least effect from neighboring segments. Vowel durations were extracted through manual labelling of the starting and ending points of each vowel token by the first author. The duration of the vowels emerged from the measurement of the interval between the starting and ending point of the vocalic part. *F1, F2*, and *F3* were normalized using the *vowels* package (Kendall & Thomas, 2018) with the Lobanov method. The normalized values were transformed into Hz in R using the formulas proposed by NORM (Thomas & Kendall, 2007). An example of the segmentation process is illustrated in Fig. 2.

LDA was employed to examine the classification of Dutch vowels in terms of Cypriot Greek listeners’ L1 categories. The analysis was conducted using the *MASS* package (Ripley et al., 2023) in R (R Core Team, 2023) (for a similar procedure, see Strange et al., 2005 and Gilichinskaya & Strange, 2010). The training and testing sets consisted of two different files that included the normalized acoustic measurements of Cypriot Greek and Dutch vowels respectively. Based on the data of the training set, we trained an L1 LDA model on mean *F1*, *F2,* and *F3* midpoint values and mean vocalic duration of Cypriot Greek vowels. The cross-validation method showed that the trained model indicated 97.9% correct classification. Therefore, the model’s high accuracy allowed us to use *F1, F2*, *F3,* and vocalic duration of Dutch vowels from the testing set and feed these values into the L1 model.

### Results

#### Production

**Fig. 3:**
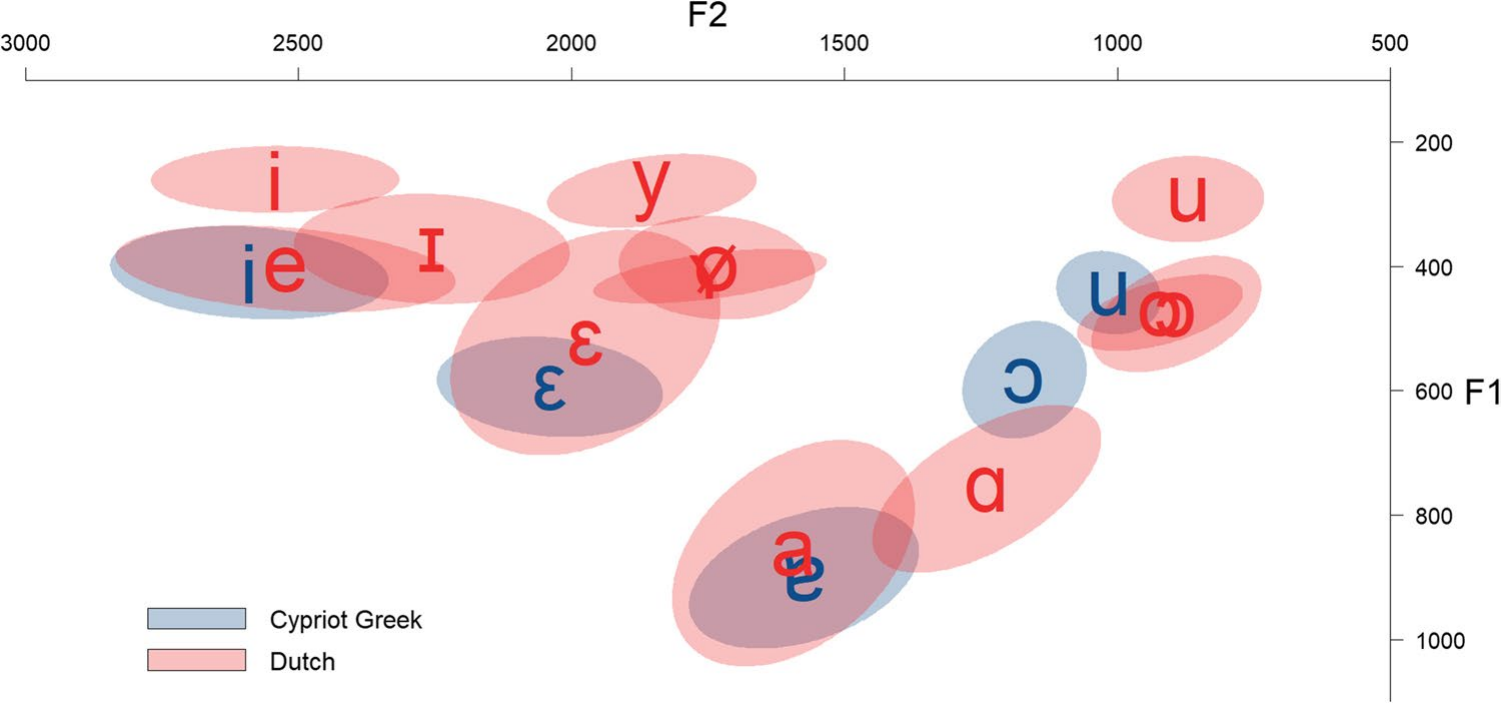
Normalized *F1 × F2* (Hz) of Cypriot Greek and Dutch vowels (scaled)

Based on the Euclidean Distance of the vowels [*d* = √[(x2 – x1)^2^ + (y2 – y1)^2^], the results of the production test show that Cypriot Greek /i/ is a very close acoustic instance of Dutch /e/ (*d* = 66) and then /i/ (*d* = 157) in terms of *F1* and *F2*. Cypriot Greek /ε/ is very close in the vowel space to Dutch /ε/ (*d* = 97). Cypriot Greek /ɐ/ and Dutch /a/ seem to be acoustically close to each other (*d* = 101), while Cypriot Greek /ɔ/ is primarily close to Dutch /ɑ/ (*d* = 188) and then /ɔ/ (*d* = 216) and /o/ (*d* = 270). Cypriot Greek /u/ is spectrally very close to Dutch /ɔ/ (*d* = 48) and then /o/ (*d* = 99). *F1 × F2* of Cypriot Greek and Dutch vowels are illustrated in Figure 3.

Among Cypriot Greek vowels, the longest duration was observed for /ɐ/. Vowels /ɔ/ and /ε/ had similar durations, while /i/ and /u/ had the shortest durations. Among Dutch vowels, /a/ had the longest duration. Dutch vowels /e o ø/, which are considered long, also had long durations. By contrast, Dutch long /i y u/ presented with short durations. The duration of Cypriot Greek vowels was closer to the duration of Dutch vowels /ɪ ɛ ʏ ɑ ɔ i y u/. Tables 1 and 2 present the average *F1, F2, F3*, and duration values of Cypriot Greek and Dutch vowels respectively as produced by L1 speakers of these languages.

**Table 1:** Average normalized *F1, F2, F3*, and duration values of Cypriot Greek vowels (scaled)

Vowels	*F1*	*F2*	*F3*	duration
i	410 (48)	2,589 (165)	2,974 (100)	123 (11)
ε	594 (52)	2,039 (133)	2,911 (78)	143 (7)
ɐ	901 (73)	1,574 (136)	2,834 (116)	156 (11)
ɔ	583 (61)	1,170 (73)	3,032 (95)	146 (9)
u	443 (43)	1,016 (62)	3,042 (121)	129 (10)

Standard deviations are shown in the parenthesis

**Table 2:** Average normalized *F1, F2, F3*, and duration values of Dutch vowels (Van der Harst, 2011). Standard deviations are shown in the parenthesis

vowels	*F1*	*F2*	*F3*	duration
a	862 (113)	1,593 (138)	2,847 (270)	278 (43)
ɑ	758 (84)	1,239 (130)	2,723 (281)	122 (24)
i	260 (33)	2,542 (141)	3,018 (135)	96 (19)
u	292 (43)	870 (87)	2,768 (260)	117 (20)
ɪ	372 (55)	2,256 (157)	2,880 (191)	105 (14)
ɔ	477 (58)	982 (97)	2,849 (286)	128 (27)
ɛ	522 (113)	1,974 (154)	2,852 (161)	139 (25)
ʏ	415 (27)	1,745 (133)	2,707 (188)	127 (20)
o	475 (38)	922 (95)	2,689 (184)	235 (46)
e	404 (43)	2,523 (194)	3,006 (159)	223 (38)
ø	402 (52)	1,734 (112)	2,536 (182)	241 (32)
y	279 (37)	1,852 (119)	2,494 (170)	93 (15)

Standard deviations are shown in the parenthesis

#### Linear discriminant analysis (LDA)

The results of LDA showed that Dutch vowels /a i u ɪ ε ʏ o ø/ were optimal responses (i.e., above chance responses in terms of a single L1 category) to Cypriot Greek vowels. Moreover, Dutch /a ɑ i u ɪ ɔ ɛ ʏ o e ø y/ were classified with the highest proportion as Cypriot Greek /ɐ ɐ i u i u ε ε ɔ ε ε i/ respectively. Apart from providing predictions about non-native vowel classification, the outcomes of the classifier can be used to develop predictions about the discrimination of non-native contrasts using the UPM framework and specifically the concept of overlapping degrees of non-native contrast members against L1 categories. In turn, these predictions are assessed through the perceptual classification test in which listeners were asked to classify the non-native vowels as their L1 categories and the discrimination test in which they discriminated particular non-native contrasts.

For the discrimination predictions, we have chosen four Dutch vowel contrasts which we anticipate to be difficult to discriminate by Cypriot Greek listeners, that is, /i – ɪ/, /ø – y/, /ɔ – o/, and /ɛ – ʏ/. We did not focus on easier contrasts (i.e., non-overlapping) as they do not create difficulties at all and are usually discriminated in an excellent manner. Under the UPM framework and based on LDA, it is expected that /ɔ – o/and /ø – y/ will be partially overlapping contrasts with moderate-to-good discrimination, while /i – ɪ/ and /ɛ – ʏ/ will be completely overlapping contrasts with poor discrimination. However, we need to consider an important parameter. Every speech sound consists of acoustic correlates or cues that distinguish it from other sounds (Chodroff & Wilson, 2020). Given that listeners pay attention to the most pertinent acoustic cues of a specific sound (Curtin et al., 2009), we assume that they may employ a single acoustic measure to classify the non-native sounds (i.e., formants or duration). Thus, we conducted a stepwise LDA to examine the power of individual acoustic measures (see Alispahic et al., 2017). We initially ran a Wilks’ lambda (Λ) test using the *klaR* package (Roever et al., 2022) from R to determine the variables that minimize Λ at an *F*-value with *p* < 0.05 and therefore improve the overall performance of the algorithm. The stepwise procedure started with the predictor that differentiated best between the vowels and included additional predictors one by one. The first step included *F2* (classification accuracy: 90.8%), the second step included *F1* + *F2* (classification accuracy: 96.7%), the third step included *F1 + F2 +* duration (classification accuracy: 97.9%), and the fourth step, which is the final model, included *F1 + F2 + F3* + duration. The results of the stepwise analysis are presented in Table 3.

**Table 3:**
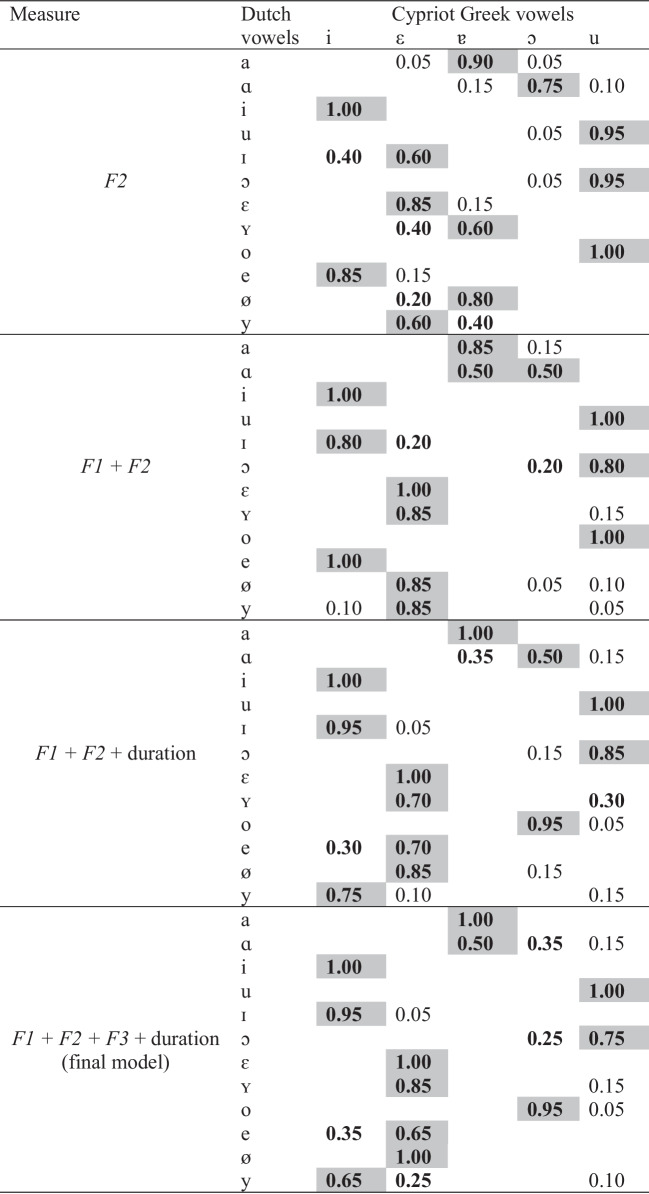
Classification results of stepwise LDA

Dark gray cells represent the L1 responses with the highest proportion. Bold cells include above-chance responses (≥ 0.20) and nonbold cells include below chance responses

According to the stepwise analysis, Dutch /ɔ/ exhibits some *F1*, *F2*, *F3,* and duration similarity to Cypriot Greek /ɔ u/, /u/, /ɔ u/, and /ɔ/, while Dutch /o/ exhibits *F1*, *F2*, *F3,* and duration similarity to Cypriot Greek /u/, /u/, /ɔ/, and /ɔ/. Therefore, /ɔ – o/ is expected to be a partially overlapping contrast with moderate-to-good discrimination. Dutch /ø/ exhibits *F1*, *F2*, *F3,* and duration similarity to Cypriot Greek /ε/, /ε ɐ/, /ε/ and /ε/, while Dutch /y/ exhibits *F1*, *F2*, *F3,* and duration similarity to Cypriot Greek /ε/, /ε ɐ/, /i ε/, and /i/. Dutch /ø – y/ is expected to be partially overlapping with moderate-to-good discrimination. Dutch /i/ exhibits *F1*, *F2*, *F3,* and duration similarity to Cypriot Greek /i/, while Dutch /ɪ/ exhibits *F1*, *F2*, *F3,* and duration similarity to Cypriot Greek /i ε/, /i ε/, /i/, and /i/. Although the final model did show that both /i – ɪ/ will be classified as Cypriot Greek /i/, partial similarity for *F1* and *F2* between the two contrast members may aid listeners to discriminate better the target contrast. This is because, if listeners rely either on *F1* or *F2* or both, they may associate the non-native vowels with different categories. Therefore, Dutch /i – ɪ/ will be discriminated in a moderate-to-good manner. However, if listeners rely on both formants and duration, the discrimination will be poor. Dutch /ɛ/ exhibits *F1, F2, F3,* and duration similarity to Cypriot Greek /ε/, while Dutch /ʏ/ exhibits *F1, F2, F3,* and duration similarity to Cypriot Greek /ε/, /ε ɐ/, /ε/, and /ε u/. Therefore, Dutch /ɛ – ʏ/ will likely present with moderate-to-good discrimination because there is some partial overlap between the two contrast members in terms of *F2* and duration. Nevertheless, if listeners rely on *F1* and *F3*, there is chance for poor discrimination.

## Perceptual study

### Methodology

#### Participants

A total number of 21 individuals (*n*_females_ = 13) participated in the study. They were Cypriot Greek speakers with an age range of 19–35 years (*M*_*age*_ = 25.62, *SD* = 4.82). All participants were born, raised, and permanently resided in Cyprus at the time of the study. They originated from moderate-income families and had never lived for more than one month in a foreign country. Participants reported excellent or very good knowledge of English and some good or basic knowledge of other languages such as French, Italian, Bulgarian, and Spanish. None of them had knowledge of Dutch. Another 10 Standard Dutch speakers (*n*_females_ = 6) with an age range of 23–38 years (*M*_*age*_ = 29.27, *SD* = 5.72) who permanently resided in the Netherlands formed the control group. All participants reported that they had never experienced any cognitive, language, or hearing problems.

#### Stimuli

The stimuli of the experiment included the 12 Dutch monophthongs embedded in an /hVb/ context, which is phonotactically possible in Dutch (Chládková & Podlipský, 2011). It is worth noting that due to final consonant devoicing, the consonant /b/ of the target word was actually pronounced as [p] by the Dutch speakers. The words were part of the Dutch carrier phrase “Hoor je <target word>”. One adult male and one adult female Dutch speaker were instructed to produce the phrases twice as naturally as possible in a quiet room. The fact that the talkers were of two different sexes did not affect the results because a pilot task showed that four Cypriot Greek listeners made very similar classifications across the two voices. The speakers’ productions were recorded using a professional audio recorder at a 44.1 kHz sampling rate and the target words were spliced out from the carrier phrase using Audacity software (Audacity Team, 2022). The output was normalized for peak intensity in Praat.

#### Procedure

The classification test was completed only by the experimental group. Each participant was tested individually in quiet rooms. The test was created in a Praat script and required participants to classify the Dutch vowels as their L1 phonetic categories. The researchers asked them to appropriately sit in front of a PC monitor and wear headphones, which were connected to the PC. During the experiment, they listened to the Dutch words including a target Dutch vowel and were asked to click on the script label, which corresponded to the most acoustically similar L1 vowel to the Dutch vowel they heard. The labels contained an orthographic transcription of the five Cypriot Greek vowels, that is, “ι”, “ε”, “α”, “ο”, “ου”. They were also asked to rate how good an acoustic exemplar the Dutch vowel was to the L1 vowel they had chosen, by selecting one of the responses from 1 (very poor) to 5 (very good). They classified a total number of 36 trials each (12 vowels × 3 repetitions) with an optional 2-min break at the midpoint. The repetitions were from the same tokens. No feedback was given to the participants during the experiment. The interval between a click and the presentation of the next trial was 500 ms. There was no time frame for participants to respond, but they were encouraged to respond as quickly as possible even if they were unsure. Before the main experiment, a familiarization test with four items was performed by the participants to ensure that they understood the requirements of the test; the items were recorded by a different speaker and were different from the test items. The test was completed within 10 min.

After the classification test, participants from both the experimental and control groups completed an AXB test in which they discriminated particular pairs of Dutch contrastive vowels. The test was scripted in Praat and included the labels “A”, “X”, and “B”. Participants listened through the headphones to a triad of the target words from the PC loudspeakers and were asked to select whether the middle vowel (X) was the same as the first (A) or the second vowel (B) by clicking on the appropriate label. Each vowel pair appeared in four possible configurations, namely, AAB, ABB, BBA, and BAA, and they discriminated a total number of 64 items (4 contrasts × 4 repetitions × 4 trials). The X token was always acoustically different (i.e., different talker) from A and B tokens to avoid a solely auditory decision (Polka, 1991). The interstimulus interval was 1s and the intertrial interval 500 ms. There was an optional 5-min break after the 32^nd^ item. There was also a four-trial familiarization test before the main test, which included different vowel contrasts from those of the main test. Each participant took approximately 15 min to complete the test.

#### Statistical analysis

Descriptive statistics were used to create the confusion matrix, which presents the classification proportion of each Dutch vowel as L1 Cypriot Greek categories. The determination of above chance categories was based on whether the percentage was greater or equal to 0.20. The analysis of the discrimination data has been conducted using treatment coding. Specifically, we fitted a binomial logistic mixed-effects model using the *lme4* package (Bates et al., 2022) to investigate differences in the discrimination accuracy between various vowel contrasts as well as differences in the discrimination accuracy of vowel contrasts between the experimental and the control groups. ACCURACY (correct (1)/incorrect (0)) was the binary dependent variable. CONTRAST (four Dutch contrasts; dummy variables: /i – ɪ/, /ø – y/, /ɔ – o/, baseline variable: /ɛ – ʏ/), LANGUAGE (dummy variable: Dutch, baseline variable: Cypriot Greek), and CONTRAST × LANGUAGE were modeled as fixed factors, while PARTICIPANT (P1-P21) and ITEM (AAB, ABB, BBA, and BAA) were modeled as random intercepts. Pairwise comparisons were conducted using the *emmeans* package (Lenth et al., 2023) with the *Tukey* method. Posthoc analyses can provide more detailed insights into the effects and interactions observed in the data and can help to identify specific differences between groups.

### Results

#### Classification test

The results indicated that Dutch /a ɑ i u ɔ ε ʏ e/ were classified as optimal responses to Cypriot Greek categories. In addition, Dutch /a ɑ i u ɪ ɔ ɛ ʏ o e ø y/ were classified with the highest proportion as Cypriot Greek /ɐ ɐ i u ε ɔ ε ε ɔ ε ε i/. These results can be compared to those provided by the final model of LDA. In terms of classifications with the highest proportion, LDA predicted accurately the classification of Dutch /a ɑ i u ε ʏ o e ø y/. Only the classification of Dutch /ɪ ɔ/ was not estimated correctly. The confusion matrix of the classification test is shown in Table 4.

**Table 4:**
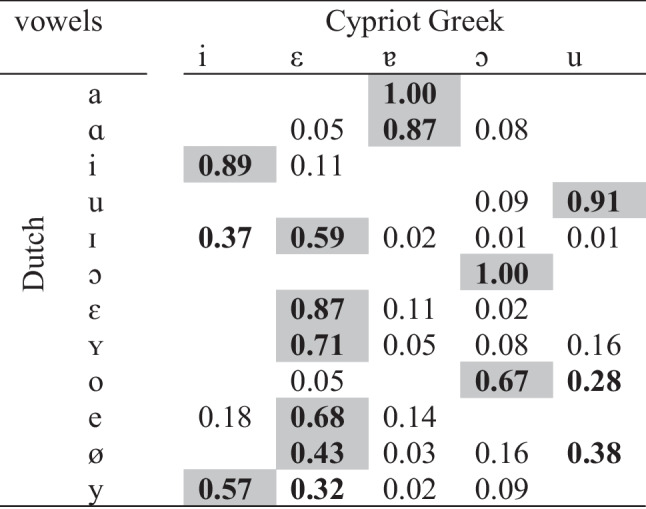
Results of the perceptual classification test

Dark gray cells represent the L1 responses with the highest proportion. Bold cells include above chance responses (≥ 0.20) and nonbold cells include below chance responses

On the basis of the classification test, the assumptions of UPM can be used to establish the predictions about the discrimination accuracy of the non-native contrasts. According to UPM, /i – ɪ/, /ø – y/, and /ɔ – o/ are partially overlapping contrasts as the classified responses share at least one above chance response, while /ɛ – ʏ/ is a completely overlapping contrast as the responses share the same above chance response. It is expected that the partially overlapping contrasts will present with moderate-to-good accuracy. Completely overlapping contrasts exhibit poor discrimination. However, there is a chance to exhibit moderate-to-good discrimination if listeners hear some acoustic differences between the sound tokens. To verify whether there was a perceived acoustic distance between /ɛ – ʏ/, we conducted a *t*-test on the goodness-of-fit ratings of both Dutch vowels classified as Cypriot Greek /ε/. The ratings revealed significant differences (*t* = 2.9, *df* = 98, *p* = 0.005), indicating that Dutch /ε/ (*M* = 3.85) was perceived as a closer exemplar to Cypriot Greek /ε/ compared to Dutch /ʏ/ (*M* = 3.16). Thus, listeners did not perceive both Dutch vowels as being equal acoustic exemplars of L1 /ε/.

#### Discrimination test

**Fig. 4:**
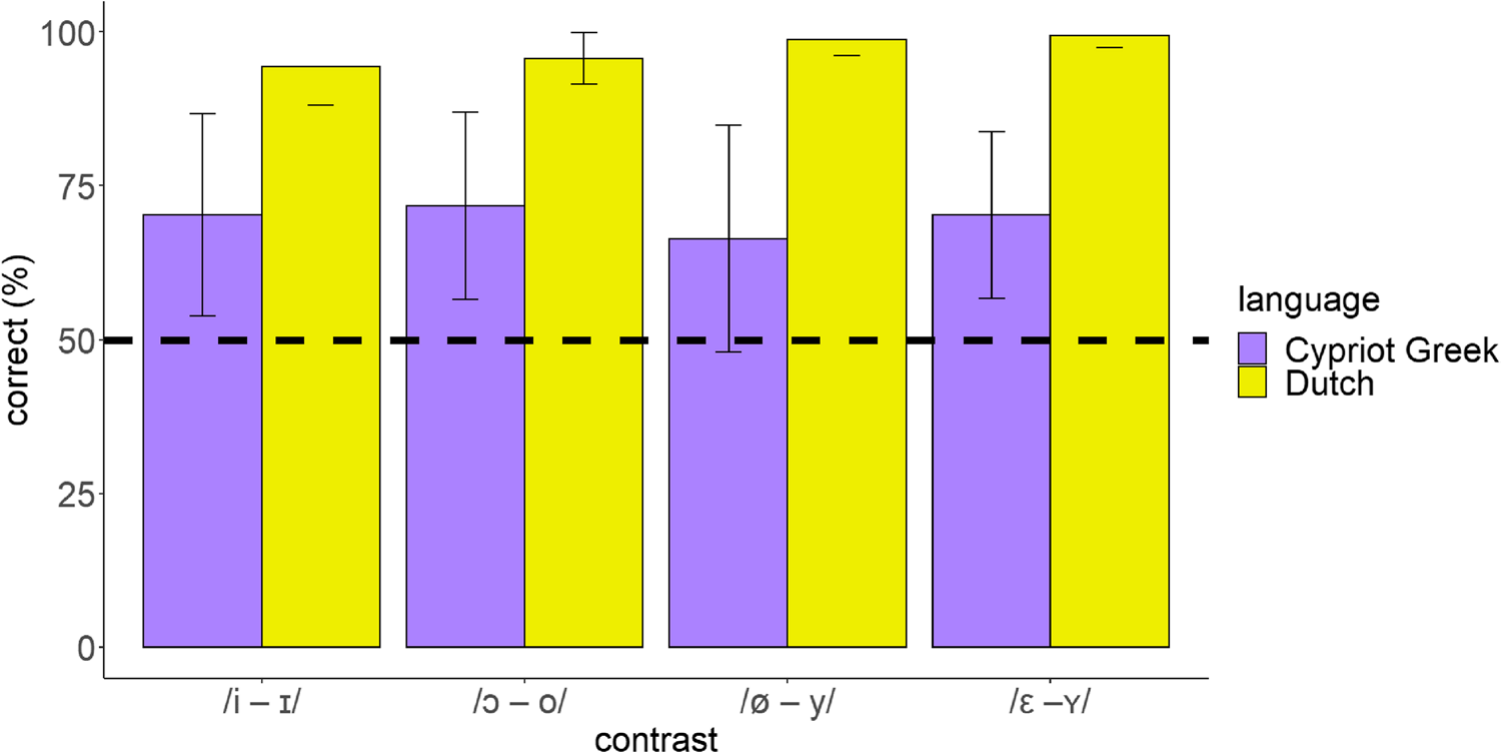
Correct discrimination of the Dutch vowel contrasts by Cypriot Greek listeners and Dutch speakers (dashed line shows chance level)

The results of the discrimination test showed that Cypriot Greek listeners discriminated the target Dutch contrasts to a moderate extent. As anticipated, the discrimination accuracy of Cypriot Greek listeners was lower compared to that of Dutch speakers. The accuracy of each Dutch contrast as discriminated by both groups is illustrated in Fig. 4. We used one-sample *t*-tests to examine whether the performance of the experimental and the control groups was above chance. The test indicated significant differences in the discrimination of each contrast by Cypriot Greek listeners and Dutch speakers, signaling above-chance performance for every contrast (Cypriot Greek: *t* = 4.08–6.84, *df* = 20, *p* < 0.001; Dutch: *t* = 22.55–79.65, *df* = 9, *p* < 0.001).

We fitted a binomial logistic mixed-effects model (dependent variable: ACCURACY; fixed factors: LANGUAGE, CONTRAST, LANGUAGE × CONTRAST; random factors: PARTICIPANTS, ITEM) to examine how various contrasts differ from each other in terms of discrimination accuracy and how the performance of Cypriot Greek listeners differs from the performance of Dutch speakers as regards the discrimination of Dutch vowel contrasts. The test reported significant differences between LANGUAGE[Dutch] and the Intercept term (LANGUAGE[Cypriot Greek], CONTRAST[/ɛ – ʏ/]), showing that the discrimination accuracy of Cypriot Greek listeners differed from that of Dutch speakers for contrast /ɛ – ʏ/. There were also significant interactions of CONTRAST[/i – ɪ/] × LANGUAGE[Dutch] and CONTRAST[/ɔ – o/] × LANGUAGE[Dutch], indicating that the discrimination of these contrasts differed from that of /ɛ – ʏ/ depending on whether the L1 of the participants was Cypriot Greek or Dutch. The results of the test are shown in Table 5.

**Table 5:** Results of the binomial logistic mixed-effects model

	Estimate	Std. Error	*z*-value	*p*-value
(Intercept)	0.889	0.208	4.275	<.0001***
contrast[/ø – y/]	0.102	0.170	0.599	0.549
contrast[/i – ɪ/]	-0.168	0.166	-1.011	0.312
contrast[/ɔ – o/]	0.043	0.169	0.255	0.798
languageDutch	2.122	0.395	5.369	<.0001***
contrast[/ø – y/]:language[Dutch]	0.164	0.541	0.304	0.761
contrast[/i – ɪ/]:language[Dutch]	1.722	0.800	2.153	0.031*
contrast[/ɔ – o/]:language[Dutch]	2.213	1.063	2.081	0.037*

Signif. codes: 0 ‘***’ 0.001 ‘**’ 0.01 ‘*’ 0.05 ‘.’ 0.1 ‘ ’ 1

To investigate whether particular contrasts differed from each other across Cypriot Greek listeners and between Cypriot Greek listeners and Dutch speakers, we used pairwise posthoc corrections on LANGUAGE × CONTRAST. The results indicated no significant differences between the discrimination accuracy of any of the Dutch contrasts for Cypriot Greek listeners. In addition, there were significant differences between Cypriot Greek listeners and Dutch speakers for each contrast; specifically, Dutch speakers discriminated the Dutch vowel contrasts more accurately than Cypriot Greek listeners. Table 6 presents the results of pairwise comparisons.

**Table 6:** Results of the CONTRAST × LANGUAGE pairwise comparisons

	Estimate	Std. Error	*z*-value	*p*-value
/ɛ – ʏ/ Cypriot Greek – /ø – y/ Cypriot Greek	-0.102	0.170	-0.599	0.999
/ɛ – ʏ/ Cypriot Greek – /i – ɪ/ Cypriot Greek	0.168	0.166	1.011	0.973
/ɛ – ʏ/ Cypriot Greek – /ɔ – o/ Cypriot Greek	-0.043	0.169	-0.255	1.000
/ø – y/ Cypriot Greek – /i – ɪ/ Cypriot Greek	0.270	0.168	1.608	0.746
/ø – y/ Cypriot Greek – /ɔ – o/ Cypriot Greek	0.059	0.171	0.344	1.000
/i – ɪ/ Cypriot Greek – /ɔ – o/ Cypriot Greek	-0.212	0.167	-1.266	0.912
/ɛ – ʏ/ Cypriot Greek – /ɛ – ʏ/ Dutch	-2.122	0.395	-5.369	<.0001 ***
/ø – y/ Cypriot Greek – /ø – y/ Dutch	-2.286	0.433	-5.276	<.0001 ***
/i – ɪ/ Cypriot Greek – /i – ɪ/ Dutch	-3.844	0.732	-5.253	<.0001 ***
/ɔ – o/ Cypriot Greek – /ɔ – o/ Dutch	-4.335	1.013	-4.279	0.001 ***

Signif. codes: 0 ‘***’ 0.001 ‘**’ 0.01 ‘*’ 0.05 ‘.’ 0.1 ‘ ’ 1

## Discussion

This study examined the perception of Dutch vowel contrasts by Cypriot Greek listeners and evaluated the predictions of cross-linguistic acoustic and perceptual similarity. The classification of non-native vowels in terms of L1 categories and the discrimination of non-native vowel contrasts were predicted using an LDA model that was trained on the formant frequencies and duration of Cypriot Greek vowels, and which was then fed with the same values for Dutch vowels. This allowed the comparison of the model’s calculations with the results of the perceptual tests for purposes of designating the role of cross-linguistic acoustic similarity in non-native speech perception. We also assessed the theoretical predictions of the UPM model based on perceptual similarity using an AXB discrimination test. The statistical analysis was conducted through a binomial mixed-effects model.

Similar to previous investigations on the perception of non-native vowels by Cypriot Greek speakers (e.g., Georgiou, 2019, 2021b), the findings of this study also revealed that multiple Dutch vowels were classified as close approximations of a single L1 vowel. This was expected, given that the target language contains a larger and more complex vowel inventory than the L1 of the listeners (e.g., see Iverson & Evans, 2007, among others). More specifically, the perceptual classification test completed by Cypriot Greek listeners revealed the following classifications: Dutch /a ɑ/ as Cypriot Greek /ɐ/, Dutch /ɪ ɛ ʏ e ø/ as Cypriot Greek /ε/, Dutch /ɔ o/ as Greek /ɔ/, Dutch /i y/ as Cypriot Greek /i/, and Dutch /u/ as Cypriot Greek /u/. In addition, the majority of Dutch vowels (i.e., /a ɑ i u ɔ ε ʏ e/) were classified as optimal responses to L1 categories, meaning that they comprised acoustically good fits to those categories. In contrast, the classification of some Dutch vowels (i.e., /ɪ o ø y/) as above chance responses in terms of more than one L1 category shows that listeners did not associate the non-native vowels with a single L1 vowel. In other words, they did not perceive these Dutch vowels as being acoustically similar to a particular Cypriot Greek vowel.

Cross-linguistic acoustic similarity measured using an LDA paradigm predicted successfully the classification of most Dutch vowels with the highest proportion within an L1 category. Specifically, it predicted the classification of 10 out of 12 vowels, namely, /a ɑ i u ɪ ɔ ɛ ʏ o e ø/. This corroborates the findings of previous studies that observed accurate prediction of most vowel categorizations (e.g., Gilichinskaya & Strange, 2010). In another study, Georgiou (2023a) found that cross-linguistic acoustic similarity could estimate accurately the classification of only seven out of 11 English vowels as Cypriot Greek categories. However, we need to take into account that the LDA model in the aforementioned study was trained only on the first two formants and the duration of vowels and not on *F3* just like our model. In terms of accurate predictions for the whole range of above chance responses, the power of acoustic similarity was confined to the classification of seven out of 12 vowels, that is, /a ɑ i u ɛ ʏ y/. Overall, cross-linguistic acoustic similarity is a good metric for the estimation of listeners’ speech sound categorization patterns. Nevertheless, as shown in a number of studies (e.g., Alispahic et al., 2017; Escudero et al., 2012), LDA might not capture the exact categorization proportion probably because linear boundaries between categories in n-dimensional spaces do not apply in human speech perception or because there could be nonlinear associations between the outcome variable and the predictors which could be grasped better by other machine-learning classifiers (e.g., for the better performance of a neural network algorithm over LDA in cross-linguistic vowel classification, see Georgiou, 2023c). Moreover, the z-score normalized input of LDA makes use of the whole vowel space, but listeners hearing a single token at a time only have vowel-intrinsic available for perceptual normalization. Therefore, this is another way LDA and human classification can be expected to differ.

In addition, acoustic similarity based on the UPM framework predicted successfully the discrimination of all Dutch vowel contrasts. As opposed to these predictions, the findings of other studies show less success. For example, Georgiou (2023b) used a similar study protocol to assess the ability of acoustic similarity to predict the accuracy of four Dutch contrasts as discriminated by Standard Modern Greek and Cypriot Greek listeners. The results demonstrated accurate prediction only for some contrasts. Again, the LDA model of the aforestated study included measures on *F1, F2*, and duration of native and non-native vowels and not on *F3*, which seems to provide more accurate calculations. Furthermore, the perceptual classification test of this study, which was based on the assumptions of the UPM model, led to similar predictions as it estimated with success the discrimination of all Dutch contrasts. Therefore, cross-linguistic perceptual similarity provides similar estimations to the acoustic similarity for the discrimination accuracy of non-native sound contrasts. The literature supports this finding. For example, Elvin et al. (2021) argued that although perceptual similarity was a better predictor of discrimination accuracy for European Spanish listeners and acoustic similarity was a better predictor of discrimination accuracy for Brazilian Portuguese listeners, both measures can reliably predict listeners’ discrimination patterns. The authors attributed this discrepancy to the differences in the sizes of the two languages’ vowel systems. Moreover, it is worth noting that despite the prediction of the final LDA model for poor discrimination of Dutch /i – ɪ/ and /ɛ – ʏ/ as they completely overlapped with Cypriot Greek /i/ and /ε/, the stepwise LDA predicted moderate-to-good discrimination because some individual measures exhibited partial overlap. Specifically, the trained model indicated some *F1* and *F2* similarity of Dutch /ɪ/ to Cypriot Greek /ε/ and some *F2* and duration similarity of Dutch /ʏ/ to Cypriot Greek /ɐ/ and /u/. Alispahic et al. (2017) also observed that a stepwise LDA predicted accurately the discrimination of non-native Dutch contrasts by adult Australian English and Peruvian Spanish listeners. Interestingly, here, listeners relied on formants to distinguish /i – ɪ/ as well as /ɛ – ʏ/, assuming that the members of the latter vowel contrast mostly differ in terms of spectral features than in terms of duration. The prioritization of spectral rather than temporal cues by Cypriot Greek listeners is also shown in previous studies (Georgiou, 2019, 2023a). Further evidence suggests that despite the variability of vowel duration across different vowels, these are distinguished from other adjacent vowels from their spectral characteristics (Hillenbrand, 2013).

As discussed before, the UPM model managed to predict the discrimination accuracy of the non-native contrasts in an accurate manner. Specifically, the classification test predicted that /ø – y/, /i – ɪ/, /ɔ – o/ will be partially overlapping contrasts, presenting with moderate-to-good discrimination and that /ɛ – ʏ/ will be a completely overlapping contrast but with the same discrimination accuracy as the previous contrasts due to perceived acoustic distance between the contrast members. Partial overlap takes place when the members of a non-native sound contrast are classified as one or more common above chance L1 responses. The discrimination results reported accuracy between 67% and 71.3% for all contrasts, which falls within the moderate range. In addition, the statistical analysis exhibited no significant differences in the discrimination accuracy of these contrasts. This is consistent with previous findings that confirm the theoretical predictions of UPM on L2 learners (e.g., Georgiou, 2021b, 2022b). The fact that Cypriot Greek listeners’ performance in the discrimination of Dutch vowel contrasts was lower compared to that of Dutch speakers shows that non-native sounds are disoriented as their acoustic features do not match those of Dutch speakers. Of course, UPM argues that listeners can potentially perceive accurately the non-native sounds across their lifespan if, for example, they receive formal phonetic training, are exposed to non-native stimuli to a great extent, live in a place where the non-native variety is dominant, etc. In this study, we cannot compare the discriminability of different types of contrasts since the overlapping degrees of the contrasts under examination were only partial. Nevertheless, further evidence is provided that partial overlap results in moderate discrimination of sound contrast members, confirming one of UPM’s predictions.

One interesting finding is that the three partially overlapping contrasts exhibited similar discrimination accuracy. Such a finding provides us with the opportunity to investigate whether partial overlap should be interpreted in an undifferentiated manner or as a continuous variable; this may have significant implications for the predictions of speech acquisition models. Flege and MacKay (2004) developed a classification overlap score, which comprises quantification of the overlapping degree for a given L2 sound contrast, in order to predict the discrimination of that contrast. The general prediction is that the larger the overlap, the poorer the discrimination accuracy. Overlap is computed by summing the smallest classification percentages when the two contrast members are classified to the same L1 category. For example, in this study, Dutch /i/ and /ɪ/ were classified as Cypriot Greek /i/ 89% and 37% of the time respectively. They were also classified as Cypriot Greek /ε/ 11% and 59% of the time respectively. Therefore, if we sum the smallest percentages, namely 37% and 11%, we get a 48%, which is the overlap score of the contrast members. By applying the same process for the vowel members of the other two partially overlapping contrasts, we get a 67% overlap score for /ɔ – o/ and a 32% overlap score for /ø – y/. The quantification of the overlap degree suggests that /ɔ – o/ would be discriminated worse than the other two contrasts due to its higher score. In turn, /i – ɪ/ would be discriminated worse than /ø – y/. However, such differences were absent. Similar discrepancies were observed by Flege and MacKay (2004). Specifically, the authors found that although English /e^ɪ^ – ε/ presented with a high classification score (i.e., 87%), it was discriminated better than other contrasts by Italian learners. In addition, the expectation for good discrimination of English /ɪ – ε/ due to its low overlap score (i.e., 35%) was not met. In Elvin et al.’s (2021) study, the differences in the perceptual overlap scores of Brazilian Portuguese /o – u/ (0.32) and /o – ɔ/ (0.70) were not accompanied by differences in the accuracy for these contrasts as discriminated by European Spanish listeners. As shown by the results of this study and previous evidence, it seems that discrimination accuracy is not directly related to the overlap degree calculated using quantification measures such as overlap scores. However, such a conclusion is just tentative and more research is needed to examine better this relationship. Still, a crucial question needs to be answered: how the similar discrimination accuracy between the three partially overlapping contrasts can be justified? Taking into account that Cypriot Greek listeners mostly rely on formant frequencies to distinguish non-native sound contrasts, the results of stepwise LDA may provide some insights into this. The second step, which includes *F1* and *F2*, shows that both of the vowel members of the contrasts /i – ɪ/, /ɔ – o/, and /ø – y/ were classified with a percentage of 80-85% in terms of L1 /i/, /ε/, and /u/ respectively and with a percentage of 15-20% in terms of other L1 categories (this would give an overlap score of 80% for /i – ɪ/ and /ɔ – o/, and 90% for /ø – y/). So, there is a similar weight of classification for the contrast members and this might explain the null differences between the discrimination accuracy of the contrasts. Such a conclusion would posit that a quantified overlap degree or the overlap score could estimate discrimination only if applied to the cues the listeners pay attention to and not to their overall perceptual patterns, at least for the contrasts investigated in this study.

The results can be discussed through the lenses of other important speech perception models. PAM makes perceptual predictions for the discrimination accuracy of non-native contrasts (i.e., contrasts of a language which a listener does not have experience with) based on cross-linguistic perceptual similarity. Using PAM’s framework, if a 70% categorization threshold is adopted, Dutch /i – ɪ/ and /ɔ – o/ would be UC contrasts, presenting with very good discrimination. Dutch /ø – y/ would be a UU contrast, which is expected to have a wide range of discrimination (poor to very good) depending on their perceptual distance; in this case, there is partial overlap in the classification of both Dutch vowels with Cypriot Greek /ε/ and therefore moderate-to-good discrimination accuracy is predicted. Dutch /ɛ – ʏ/ would be a CG contrast with moderate to very good discrimination since there is perceived phonetic distance between the two sounds. PAM assumes that CG contrasts can have similar discrimination accuracy to UC and in some circumstances, UU contrasts. This is confirmed by the similar discrimination accuracy of all contrasts, which is also close to a large extent to the discrimination predicted by the framework of UPM. In addition, the results can be discussed using the framework of another widely used speech model, namely L2LP, of which the predictions rely on cross-linguistic acoustic similarity. On the basis of the LDA classifications, although Dutch /i – ɪ/ and /ε – ʏ/ seem to be part of the New Scenario, these belong in fact in the Subset Scenario because the stepwise LDA showed that *F1* and *F2* of Dutch /ɪ/ were classified as both Cypriot Greek /i/ and /ε/ and that *F1, F2, F3*, and duration of Dutch /ʏ/ were classified as Cypriot Greek /ε/, /ε ɐ/, /ε/, and /ε u/ respectively. Similarly, Dutch /ɔ – o/ and /ø – y/ would also result in the Subset Scenario as one of the two members was classified as two Cypriot Greek categories. As the overlap between the Dutch vowel members is partial (and neither complete nor nonexistent, see Subset Difficult and Subset Easy in Elvin et al., 2021), moderate-to-good discrimination is expected. This is consistent with the discrimination accuracy estimated by UPM as well. Concluding, both PAM and L2LP can successfully predict the discrimination of Dutch vowel contrasts on the basis of perceptual and acoustic similarity respectively and their results agree with those of UPM.

## Conclusion

This study designated the important role of acoustic and perceptual cues in predicting sound discrimination patterns of non-native listeners. In addition, the predictions of the UPM model for moderate discrimination of the members of challenging partially overlapping contrasts were confirmed even though the current study did not investigate contrasts with complete overlap (in which there is no perceived acoustic distance between the non-native sounds) or no overlap. Future examinations investigating the discriminability of members of completely overlapping or non-overlapping contrasts can provide further insights into the perceptual patterns of listeners from an understudied L1 background in target languages with more complex vowel inventories. Such studies can investigate to a greater extent the role of cross-linguistic acoustic similarity in non-native speech perception apart from providing further support for the assumptions of UPM.

## Data Availability

Data are available on request.
